# Towards Imaging Tau Hyperphosphorylation: Is DYRK1A a Potential Target for Imaging Hyperphosphorylation of Tau? Molecular Modeling Assessment and Synthesis of [^125^I]Radioiodinated DYRK1A Inhibitor

**DOI:** 10.3390/molecules30050990

**Published:** 2025-02-21

**Authors:** Cayz G. Paclibar, Deanna M. Schafer, Agnes P. Biju, Fariha Karim, Stephanie A. Sison, Christopher Liang, Shamiha T. Ahmed, Jogeshwar Mukherjee

**Affiliations:** Preclinical Imaging, Department of Radiological Sciences, University of California-Irvine, Irvine, CA 92697, USA; cgpaclib@uci.edu (C.G.P.); dmschafe@uci.edu (D.M.S.); apbiju@uci.edu (A.P.B.); fkarim1@uci.edu (F.K.); sisonsa@uci.edu (S.A.S.); liangc@uci.edu (C.L.); shamihaa@uci.edu (S.T.A.)

**Keywords:** [^125^I]IPPI, [^125^I]KuFal184, DYRK1A, post-mortem human Tau, Alzheimer’s disease, down syndrome, autoradiography

## Abstract

Dual specificity tyrosine-phosphorylation regulated kinase 1A (DYRK1A), a phosphorylation kinase, is localized within the central nervous system and is linked to hyperphosphorylation of Tau. Imaging of DYRK1A may provide an earlier biomarker for Tauopathies, including Alzheimer’s disease (AD). We have used Chimera-Autodock to evaluate potential molecules for binding to the binding site of DYRK1A. Five molecules, 10-bromo-2-iodo-11*H*-indolo[3,2-c]quinoline-6-carboxylic acid (4E3), 10-iodo-11*H*-indolo[3,2-c]quinoline-6-carboxylic acid (KuFal184), harmine, 6-(fluoro-3-(1H-pyrrolo[2,3-c]pyridin-1-yl)isoquinolin-5-amine (MK-6240), and 6-iodo-3-(1H-pyrrolo[2,3-c]pyridine-1-yl)isoquinoline (IPPI), were found to have binding energies of −10.4, −10.1, −9.0, −9.1, and −9.4 kcal/mole, respectively. Two molecules, 4E3 and KuFal184, were selective for DYRK1A, while harmine also had a monoamine oxidase A affinity, and MK-6240 and IPPI had affinity for Tau. Tau present in the brain slices of AD subject were labeled with [^125^I]IPPI. KuFal184 had no effect on the binding of [^125^I]IPPI, suggesting the absence of binding overlap of the two molecules. MK-6240, a known Tau agent was, however, able to compete with [^125^I]IPPI. The binding energies of harmine, MK-6240, and IPPI for the DYRK1A site suggest affinities of approximately 80–100 nM, which is insufficient to serve as an imaging agent. The higher affinity of KuFal184 (6 nM for DYRK1A) suggested that [^125^I]KuFal184 may be a potential imaging agent. Electrophilic radioiodination was used to synthesize [^125^I]KuFal184 in modest yields (25%) and high radiochemical purity (>95%). Preliminary binding studies with [^125^I]KuFal184 in AD brain slices showed some selectivity for cortical grey matter regions containing Tau.

## 1. Introduction

Hyperphosphorylation of Tau at the various serine (Ser), threonine (Thr), and tyrosine (Tyr) residues is carried out by several protein kinases [1]. They are broadly classified into proline-directed protein kinases (PDPK, targeting phosphorylation of Ser and Thr, which precede a proline residue, i.e., Ser/Thr-Pro) and includes four different kinases, namely glycogen synthase kinase-3 (GSK3), cyclin-dependent protein kinase-5 (Cdk5), p38 mitogen-activated protein kinases (P38 MAPK), and dual specificity tyrosine-phosphorylation regulated kinase 1A (DYRK1A) (Table 1). The non-proline directed kinases (non-PDPK) include the microtubule affinity-regulating kinase 4 (MARK4), the tyrosine protein kinases (Src, c-Abl, Fyn), and the Rho-associated kinase (ROCK), which have been discussed in detail [1]. These kinases are ubiquitously expressed because of their roles in various other cellular functions. Efforts to target the kinases for the development of therapies for Alzheimer’s disease (AD) have not yet been successful [1]. Over the last several years, efforts have evaluated the use of plasma biomarkers for AD [2]. The phosphorylated Tau (p-Tau) present in the cerebrospinal fluid (CSF) and blood plasma are being measured for p-Tau181, 217, 231, and 235 in plasma and CSF and used for the staging of AD and Down’s syndrome (DS) [3,4,5,6,7,8] (Table 1). More recently, p-Tau181 and 217 have been measured as blood markers in DS [9]. These four phosphosites appear to be driven by GSK3, Cdk5, and P38 MAPK, while the phosphorylation contribution of each of the kinases is not known. In vivo Tau-positron emission tomography (PET) brain imaging and p-Tau plasma/CSF measures have been correlated, suggesting the value of p-Tau plasma/CSF measures [3].

In vivo imaging of kinases in the brain may serve as another biomarker for the assessment of hyperphosphorylation. However, no major effort has gone into the development of molecular imaging agents that may be able to track kinases or their activities. There are several challenges in pursuing kinases, including their ubiquitous presence, cytoplasmic or membrane-bound, high affinity and selective small molecules, blood brain barrier permeability, concentration in brain regions, measurement of enzyme activity rather than concentration, and other considerations.

**Figure 1:**
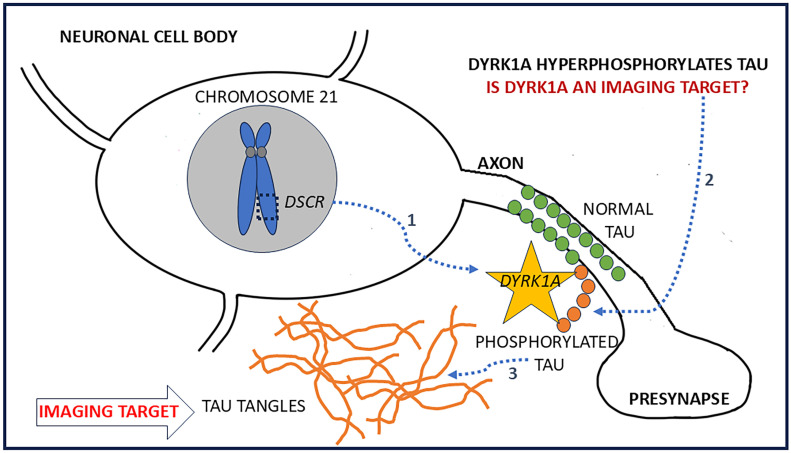
Schematic showing chromosome 21 with the Down’s syndrome critical region (DSCR) highlighted. Expression of DYRK1A is regulated by DSCR and is upregulated in Down’s syndrome (#1 blue dotted line). Membrane-bound DYRK1A may play a role in the hyperphosphorylation of Tau (#2 blue dotted line), resulting in the formation of neurofibrillary tangles (#3 blue dotted line). (Orange star: DYRK1A kinase; Green circles: normal Tau; Orange circles: phosphorylated Tau; Orange lines: Tau tangles).

DYRK1A belongs to a family of protein kinases known as DYRKs, responsible for neuronal development and several biological processes [10,11]. DYRK is a highly regulated kinase whose dysregulation may lead to cancer, diabetes, and neurological diseases [12,13]. Down syndrome chromosome 21 has the DS critical region (DSCR) region, which regulates the higher expression of DYRK1A [14,15] (Figure 1). DYRK1A has been associated with the accumulation of β amyloid peptides and Tau phosphorylation. In humans, intracellular DYRK1A has been reported to be associated with the cytoskeleton as well as cytosolic and nuclear fractions and phosphorylates Tau, resulting in Tau tangles [16,17]. Hyperphosphorylation of Tau at Ser202, Ser404, and Thr212 has been indicated with DYRK1A dysregulation and likely leads to the formation of Tau tangles [1,12,16]. This membrane-bound DYRK1A may be a potential in vivo imaging target. More importantly, measurement of its kinase activity may provide a means of earlier abnormalities in AD since the hyperphosphorylation activity precedes the formation of Tau tangles.

**Figure 2:**
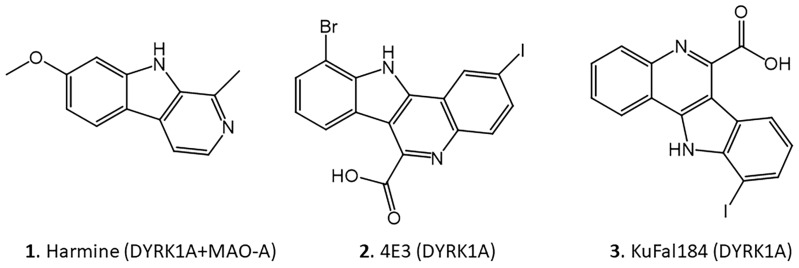
Chemical structures of compounds with reported binding profiles. **1**. Harmine, binds to DYRK1A and MAO-A; **2**. 10-bromo-2-iodo-11*H*-indolo[3,2-c]quinoline-6-carboxylic acid (4E3) binds to DYRK1A; **3**. 10-iodo-11*H*-indolo[3,2-c]quinoline-6-carboxylic acid (KuFal184) binds to DYRK1A.

Recent efforts have focused on the development of small molecule inhibitors for DYRK1A as potential therapeutics for various diseases [18,19]. Using the backbone structure of harmine (**1**, Figure 2), efforts have been made to develop more selective DYRK1A drugs, since harmine binds to both monoamine oxidase A (MAO-A) and DYRK1A [20]. A series of 10-iodo-11H-indolo[3,2-c]quinoline-6-carboxylic acid derivatives have been found to be potent and selective DYRK1A inhibitors [21]. A new drug, 10-bromo-2-iodo-11*H*-indolo[3,2-c]quinoline-6-carboxylic acid (4E3, **2**, Figure 2), has been developed that is selective for DYRK1A and does not inhibit MAO-A activity [22]. One of the most effective molecules with a 10-iodo substituent has been found (10-iodo-11*H*-indolo[3,2-c]quinoline-6-carboxylic acid, (KuFal184, **3**, Figure 2) [21,23].

Since pharmacological approaches towards inhibition or modulation of Tau phosphorylation are being pursued for the various kinases [1,20,21,22,23,24], imaging of the kinases in the central nervous system (CNS) will be very valuable, as imaging biomarkers for neurofibrillary tangles (NFT) in AD. Although it has been over 15 years since the role of the kinases as pharmacological targets were raised [24], to the best of our knowledge, no successful imaging effort of developing imaging agents for Tau phosphorylation kinase has yet been reported.

The goals of this paper are: 1. Use molecular modeling to evaluate the binding of the compounds in Figure 2 to DYRK1A and compare them to known Tau NFT binding agents, 6-(fluoro-3-(1H-pyrrolo[2,3-c]pyridin-1-yl)isoquinolin-5-amine (MK-6240) and 6-iodo-3-(1H-pyrrolo[2,3-c]pyridine-1-yl)isoquinoline (IPPI) [25,26]; 2. Evaluate the effects of KuFal184 on the binding of [^125^I]IPPI in postmortem AD brain slices; 3. Synthesize iodine-125-labeled [^125^I]KuFal184 for in vitro studies of DYRK1A, and 4. Preliminary assess [^125^I]KuFal184.

## 2. Results

### 2.1. DYRK1A Molecular Modeling

**Figure 3:**
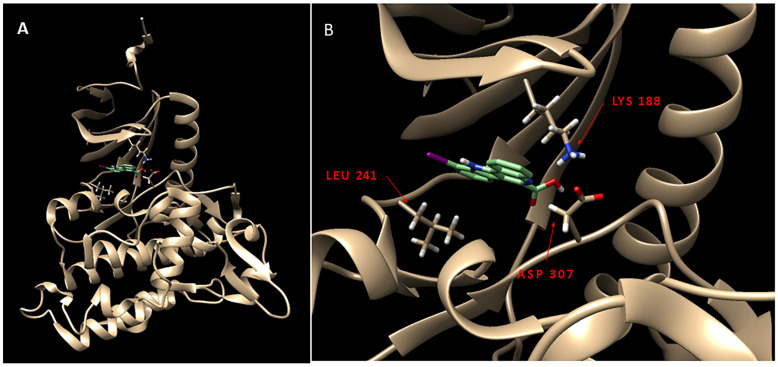
(**A**). DYRK1A (PDB 4YLL [18] protein structure used for binding energy measurements of various molecules; (**B**). Interaction of KuFAL184 at the binding site of DYRK1A. Three residues of DYRK1A binding site have been highlighted in red, including Leu241, Asp307, and Lys188. The high affinity of KuFal184 is driven by the “salt bridge” interaction between the carboxylate group in KuFal184 and Lys188.

Using the 3-structure of DYRK1A available in the Protein Data Bank (PDB) database (PDB 4YLL [21,22]), binding studies of the molecules are shown in Figure 2. Since harmine exhibited affinity for DYRK1A but also had high affinity for monoamine oxidase-A, efforts were made to use the harmine backbone structure to develop selective DYRK1A molecules. A research group utilized a co-crystallized ligand, 4E3 (**2**, Figure 2), as a positive control in experiments using a Chimera-AutoDock Vina software (version 1.17.3; 2023) that bound this ligand to DYRK1A. The reported binding energy was −9.9 Kcal/mol, showing that 4E3 interacted well with DYRK1A [22]. Using Chimera-Autodock, our measurement of binding energy for 4E3 was −10.3 kcal/mol (Table 2), in close agreement with reported values. The related quinoline-6-carboxylic acid derivative KuFal184 was investigated (**3**, Figure 2) [21]. KuFal184 was amongst the most potent derivatives with high selectivity towards DYRK1A (Figure 3). The binding affinity of KuFal184 was 6 nM for DYRK1A, while harmine, the nonselective DYRK1A inhibitor, was over 10 times weaker (Table 2). Harmine also binds to MAO-A with high affinity of 5 nM [20,27]. The previous papers support that both 4E3 and KuFal184 have high affinity towards DYRK1A. Using the same software, AutoDock Vina, our analysis performed on 4E3, KuFal184, and harmine exhibited consistent binding energies of −10.3, −10.1, and −9.0 kcal/mol, respectively.

Figure 3B shows the interaction of KuFal184 with the DYRK1A binding site. Three important residues of the DYRK1A binding site were highlighted, including Leu241, Asp307, and Lys188. The high affinity of 4E3 and KuFal184 was driven by the “salt bridge” interaction between the carboxylate group in KuFal184 and Lys188. Lack of this carboxylate in harmine and an inability to form a salt bridge with Lys188 reduced its affinity for DYRK1A. The presence of a halogen (iodine or bromine), as in 4E3 and KuFal184, perhaps increases binding in a hydrophobic pocket in the vicinity of Leu241. Design of compounds that take these features in consideration are currently underway so that optimal brain-permeable, selective DYRK1A inhibitors may be evaluated in vitro and in vivo [23].

**Figure 4:**
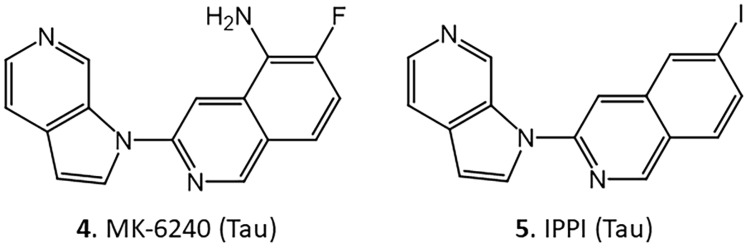
Chemical structures of Tau binding agents. **4**. 6-(fluoro-3-(1H-pyrrolo[2,3-c]pyridin-1-yl)isoquinolin-5-amine (MK-6240), binds to Tau; **5**. 6-iodo-3-(1H-pyrrolo[2,3-c]pyridine-1-yl)isoquinoline (IPPI), binds to Tau.

**Figure 5:**
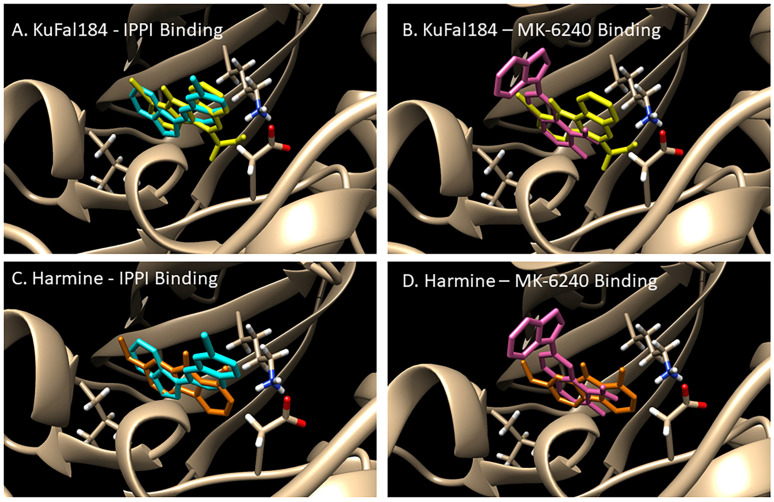
DYRK1A (PDB 4YLL [28]) Chimera-Autodock molecular models: energy-minimized comparative chemical structures in the binding site. (**A**). Energy-minimized comparison of KuFal184 and IPPI at the DYRK1A binding site; (**B**). Energy-minimized comparison of KuFal184 and MK-6240 at the DYRK1A binding site; (**C**). Energy-minimized comparison of harmine and IPPI at the DYRK1A binding site; (**D**). Energy-minimized comparison of harmine and MK-6240 at the DYRK1A binding site.

A certain amount of structural similarity between harmine and the Tau imaging agents [^18^F]MK-6240 and [^125^I]IPPI has been observed (Figure 2 and Figure 4). After analysis with AutoDock Vina, these molecules expressed similar binding energies to harmine towards DYRK1A (−9.1 and −9.4 kcal/mole for MK-6240 and IPPI, respectively, Table 2). Figure 5 shows the overlay of IPPI with KuFal184 and harmine (Figure 5A,C) and MK-6240 overlaid with KuFal184 and harmine (Figure 5B,D). IPPI appeared to have greater correspondence in the DYRK1A binding site compared to MK-6240. The interaction of Asp307 with the amino group likely changed the orientation of MK-6240 compared to IPPI, which lacks the amino group.

**Table 2:** DYRK1A and Tau binding energies of agents.

Compound	Name	^a^ DYRK1A (Kcal/mol)	^c^ Affinity DYRK1A	^d^ Tau Sites 1, 2, 3, 4 (Kcal/mol)	MAO-A
**1**	4E3	−10.3 (−9.9) ^b^	12 ^d^	−5.8, −6.8, −7.5, −6.5	NA
**2**	KuFal184	−10.1	6 nM ^d^	−5.8, −8.0, −7.4, −6.6	NA
**3**	Harmine	−9.0	70 nM ^e^	−7.4, −7.0, −6.8, −6.6	5 nM ^f^
**4**	MK-6240	−9.1	NA	−8.7, −8.8, −8.3, −7.3	>10 μM ^g^
**5**	IPPI	−9.4	NA	−7.6, −8.1, −8.2, −7.5	>10 μM ^h^

^a^ Binding energies were measured using Chimera-Autodock on DYRK1A; ^b^ [22]; ^c^ Reported binding affinities for DYRK1A; ^d^ [18,21] IC_50_; ^e^ [20]; IC50; ^f^ MAO-A IC_50_ = 5 nM and DYRK1A 32 nM [27]; ^g^ [29]; ^h^ [25].

### 2.2. In Vitro [^125^I]IPPI Binding

**Figure 6:**
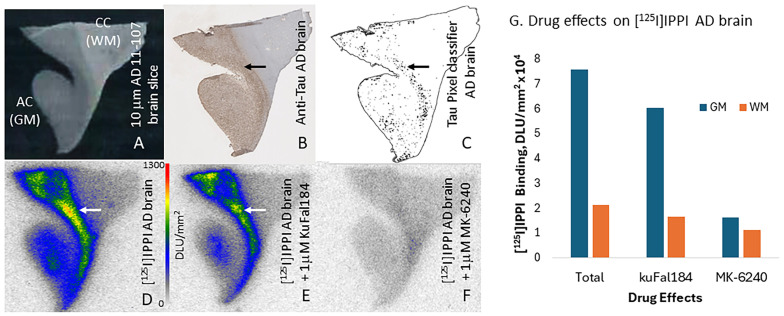
Drug effects on [^125^I]IPPI binding in AD brain: (**A**). AD (subject 11-107) brain slice (10 μm thick) showing anterior cingulate AC (GM) and corpus callosum CC (WM); (**B**). Adjacent slice (AD 11-107) anti-Tau immunostained (arrow showing Tau); (**C**). Tau pixel classifier using QuPath applied to immunostained section. Tau (shown by arrow) are identified; (**D**). Total binding of [^125^I]IPPI in AD 11-107) (arrow shows binding to Tau; autoradiography scale bar: 0–1300 digital light units DLU)/mm^2^); (**E**). Competition of [^125^I]IPPI with KuFal184 (1 μM) (arrow shows little change of binding to Tau); (**F**). Competition of [^125^I]IPPI with MK-6240 (1 μM); (**G**). Plot showing drug effects in AD-11-107 in GM and WM.

Because of the significant binding energy measured for IPPI for DYRK1A (−9.4 Kcal/mole, Table 2), in vitro studies and competition with KuFal184 were carried out. Anterior cingulate from AD subjects was used for the evaluation of [^125^I]IPPI binding. The brain slice (Figure 6A) consisted of the anterior cingulate (AC; gray matter, GM) and corpus callosum (CC; white matter, WM). Figure 6D shows the binding of [^125^I]IPPI to the GM regions in AD 11-107 with significantly lower binding in WM. The [^125^I]IPPI GM/WM binding ratio of 3.6 was measured. In the presence of KuFal184 (1 μM; Figure 6E), a 20% reduction in the binding of [^125^I]IPPI was observed in the GM (Figure 6G); however, the GM/WM ratio remained at 3.6, suggesting little effect of KuFal184 on the binding of [^125^I]IPPI. This suggests that [^125^I]IPPI is not binding at the same DYRK1A site as KuFAl184. As expected, a significant decrease in the binding of [^125^I]IPPI was observed in the presence of MK-6240 (Figure 6F). Binding in the GM was reduced >80%, and the GM/WM ratio was found to be 1.4, confirming the overlap of IPPI and MK-6240 [25]. The adjacent anti-Tau immunostained (IHC) brain section of AD 11-107 indicated the presence of abundant Tau (Figure 6B). Areas of Tau-positive IHC were identified using the QuPath pixel classifier ([26] Figure 6C), which was consistent with [^125^I]IPPI binding (Figure 6D).

### 2.3. [^125^I]KuFal184 Radiosynthesis

**Figure 7:**
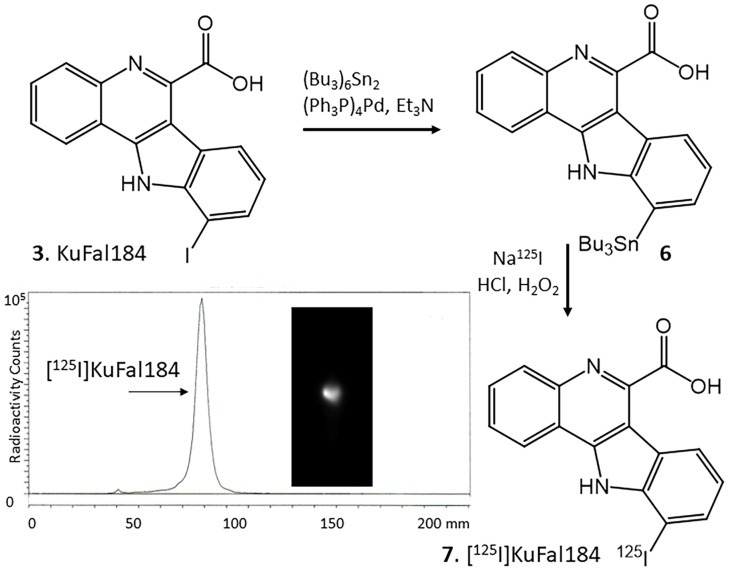
Radiosynthesis of [^125^I]KuFal184: KuFal184, **3** was refluxed with bis(tributyltin) in the presence of tetrakis(triphenylphosphine)palladium(0) for 24 h to provide tributyltin precursor, **6**. Tin precursor **6** was reacted with sodium [^125^I]iodide under oxidative conditions using hydrogen peroxide to provide [^125^I]KuFal184, **7**. Radioactive thin layer chromatogram of [^125^I]KuFal184 shows a predominant peak with purity of >95%.

Since KuFal184 contains an iodine substituent, an iodine-125 labeled analog of KuFal184 would be possible. This [^125^I]KuFal184 would then serve as a direct radiolabeling method for in vitro studies of DYRK1A. The tributyltin precursor **6** of KuFal184 was prepared as shown in Figure 7. Treatment with bistributyl tin in the presence of palladium catalyst in triethylamine provided the desired tributyl tin precursor. The radioiodination of tributyltin derivative **6** was carried out using iodine-125 sodium iodide, similar to our previously described procedures [30,31,32]. Using the molar activity of the commercial no-carrier-added [^125^I]sodium iodide, the molar activity of [^125^I]KuFal184 was estimated to be approximately 90 TBq/mmol. The radiochemical yield of [^125^I]KuFAl184 was approximately 25%. RadioTLC of the final product showed a radiochemical purity of >95% (Figure 7).

### 2.4. In Vitro [^125^I]KuFal184 Binding

**Figure 8:**
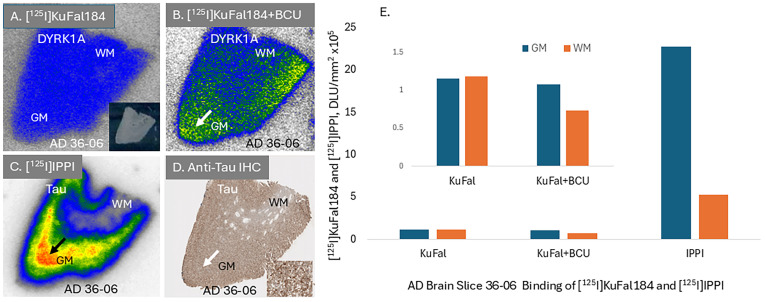
Binding of [^125^I]KuFal184 and [^125^I]IPPI binding in AD brain slice: (**A**). AD (subject 36-06) brain slice (10 μm thick temporal cortex) showing uniform non-selective binding of [^125^I]KuFal184 in GM and WM regions (inset shows scan of the brain slice); (**B**). Adjacent slice (AD 36-06) showing binding of [^125^I]KuFal184 in GM and WM in the presence of 10 μM 1,3-bis(4-cyanophenyl)urea (BCU). Higher GM binding was observed (arrow) with a GM/WM ratio of 1.46. (**C**). Total binding of [^125^I]IPPI in AD 36-06, (arrow shows binding to Tau in GM), with a GM/WM ratio of 4.35; (**D**). Anti-Tau immunostained (IHC) AD 36-06 section showing Tau (arrow), and inset shows magnified view; (**E**). Plot showing binding of [^125^I]KuFal184 (with and without BCU) and [^125^I]IPPI in GM and WM of AD 36-06 (inset figure shows rescaled *y*-axis for [^125^I]KuFal184 binding).

Preliminary in vitro binding studies using [^125^I]KuFal184 were carried out in AD brain slices. High levels of nonspecific binding are seen in the entire brain slice, including both GM and WM regions with no selectivity (Figure 8A). Since [^125^I]KuFal184 is a carboxylic acid derivative, there are concerns about its ability to cross the cell membrane. To assist in membrane transport, permeability across membranes of carboxylic acid derivatives has been reported by using transmembrane anion transporters, such as 1,3-diphenyl ureas [33]. Our preliminary studies using 1,3-bis(4-cyanophenyl)urea (BCU) did alter the levels of binding in both GM and WM regions, with GM showing some selective binding of [^125^I]KuFal184 (Figure 8B). A GM/WM ratio of 1.46 was observed in the AD brain slice. Thus, BCU appears to assist in more selective localization of [^125^I]KuFal184 in the GM regions where more DYRK1A levels are expected. Adjacent brain slice of the same subject was also treated with [^125^I]IPPI and high binding in the GM was observed (GM/WM ratio of 4.35, Figure 8C). The binding of [^125^I]IPPI correlated with anti-Tau IHC, as shown in Figure 8D.

## 3. Discussion

There is continued interest in the development of new and optimal hyperphosphorylated Tau imaging agents to address potential shortcomings of existing ones and develop new agents for different stages of Tauopathies [34,35,36,37]. The availability of such unique imaging agents may enable studies related to neurotransmitter anomalies in the AD brain [38,39]. Antibody treatment approaches to AD are also likely to gain from small molecule imaging of AD neuropathologies [28,40]. Studies suggest that p-Tau can quantify longitudinal changes in Tau pathology, identify neurodegeneration, and predict AD progression [1,2,3,4,5,6,7,8]. Consistent with CSF measures of p-Tau, current data suggest that in addition to Aβ imaging, NFT imaging can play an essential role in clinical studies for the evaluation of disease progression.

Among the various protein kinases listed in Table 1, DYRK1A plays a role in various diseases and may have a particular role in Down’s syndrome AD (DSAD). A successful in vivo imaging agent for the kinases has the potential to be an earlier biomarker because of its ability to generate p-Tau, which leads up to NFT (Figure 1). Chromosome 21, which contains the DSCR region expresses 1.5 times greater DYRK1A, resulting in greater p-Tau, and thus NFT [9]. There is now an increasing effort on drug development for DYRK1A for potential therapeutic applications in various disorders. Harmine has been shown to have a moderate affinity for DYRK1A and MAO-A. Harmine and its analogs have been previously used for imaging of MAO-A [28,40]. However, because of its affinity for DYRK1A as well, it has served as a basis for the design of high-affinity DYRK1A molecules [20,21,22,23]. Two such molecules, namely 4E3 and KuFal184, are shown in Figure 2.

Chimera-Auto Dock was used to evaluate the nature of binding of harmine, 4E3 and KuFal184 molecules to DYRK1A (PDB 4YLL [18]) and also compare them with the binding of two Tau imaging agents, [^18^F]MK-6240 and [^125^I]IPPI. Table 2 shows the binding energies of the five molecules for DYRK1A in the order of: 4E3 > KuFal184 > IPPI > MK-6240 > harmine. The binding energies of 4E3 and KuFal184 are consistent with previous findings and high affinity of these two molecules to DYRK1A [21,22]. By design, the two molecules incorporate the carboxylate group, which forms a strong salt bridge interaction with Lys188 (Figure 3B). The findings of IPPI and MK-6240 were not expected since these two agents are known to be Tau binding agents and have been shown to bind well at the 4 different binding sites of Tau fibril using Chimera-AutoDock [25]. Harmine exhibited poor binding to Tau fibrils, similar to 4E3 and KuFal184 (Table 2). In order to further evaluate DYRK1A binding of MK-6240 and IPPI, energy minimized overlays of the two molecules were carried out with harmine and KuFal184. The binding pattern of MK-6240 was significantly different compared to harmine and KuFAl184 (Figure 5B,D). This may be due to an amino group of MK-6240 orienting away from Lys188 and moving towards Asp307. In the case of IPPI, there was greater overlap, although the iodine atom in IPPI had moved to a different position, compared to KuFal184. Our previously developed smaller Tau imaging agents, [^125^I]INFT [30] and [^125^I]ISAS [32], had significantly weaker binding profiles, in agreement of lacking a “harmine-like” backbone.

The binding energies of harmine, IPPI, and MK-6240 to DYRK1A are approximately similar and based on the binding affinity of harmine for DYRK1A at 80 nM, the binding affinity of MK-6240 and IPPI may also be in this ballpark. However, this will have to be ascertained by actual binding assays of MK-6240 and IPPI for DYRK1A. Because of the significantly higher affinity of harmine for MAO-A sites, it may be surmised that in vivo and in vitro experiments with [^11^C]harmine [41] and [^18^F]fluorinated harmine derivatives [39] likely describe the distribution of MAO-A and not DYRK1A. Both in vivo PET experiments and in vitro autoradiographic experiments require radioligands with high binding affinity, typically below 10 nM.

Both MK-6240 and IPPI exhibit nanomolar affinities for human Tau [25,29]. This is reflected in their low binding energies for the four Tau binding sites (Table 2), whereas 4E3, KuFal184, and harmine displayed higher energies. Using AD brains, autoradiographic experiments with [^125^I]IPPI binding was not affected by KuFal184 (1 μM), whereas it was significantly blocked (>80%) by the same concentration (1 μM) of MK-6240 (Figure 6). This may suggest that IPPI (and MK-6240) do not bind to the DYRK1A binding site and are thus likely selective Tau imaging agents. However, additional experiments are needed to ascertain this due to the potential membrane permeability issues of KuFal184.

For autoradiographic studies of DYRK1A, an imaging agent labeled with a radioisotope would be required. Coincidently, KuFal184 possesses an iodine atom, which could be radiolabeled. We thus prepared [^125^I]KuFal184 by first preparing the corresponding pure tributyltin derivative, which was then radiolabeled with electrophilic iodine-125. The pure radiochemical product, [^125^I]KuFal184, was successfully prepared. In vitro binding studies in AD brains are currently underway. Our preliminary in vitro findings suggest that there is likely a need to enhance the membrane permeability of [^125^I]KuFal184 in order to increase binding to the GM regions and reduce the nonspecific binding. Thus, membrane permeability by using diphenyl urea such as BCU in the binding assay incubations resulted in some increased selectivity of [^125^I]KuFal184 for the GM regions (GM/WM = 1.46). The GM regions were confirmed by IHC and [^125^I]IPPI to contain Tau (Figure 8B–D). However, further experiments are required using additional AD brain samples and competition experiments using other known drugs (such as harmine) to ascertain if the binding of [^125^I]KuFal184 is indeed to DYRK1A.

The presence of the necessary carboxylate functionality in both 4E3 and KuFal184 for DYRK1A binding is a complicating factor for both in vitro and in vivo studies. There are now reports on efforts to prepare more brain-permeable derivatives of KuFAl184 [18,23]. These new derivatives may provide greater membrane permeability and offer potential alternatives for radiotracer development.

## 4. Materials and Methods

### 4.1. General Methods

General methods were similar to those described previously [25,26,27,29]. KuFal184 (Dyrk1A-IN-5) was purchased from AABlocks LLC, San Diego, CA, USA, and MK-6240 and BCU were purchased from 1ClickChemistry, Tinton Falls, NJ, USA. [^125^I]IPPI was prepared as described previously [25].

### 4.2. Molecular Modeling

Using Chimera-Autodock (version 1.17.3; 2023) as previously described for our experiments with Tau ligands [25], DYRK1A (PDB 4YLL [28]) was used to model the various molecules for DYRK1A binding energy calculations. The bound ligand (10-bromo-substituted 11H-indolo[3,2-c]quinolone-6-carboxylic acid) was first removed from the binding site of PDB 4YLL. This DYRK1A was used for molecular modeling of 4E3, KuFal184, harmine, MK-6240, and IPPI using previously reported Chimera-Autodock methods [25]. Binding energies were reported as kcal/mole.

### 4.3. Radiosynthesis

To a solution of KuFal184, **3** (4 mg; 10 μmol) in anhydrous triethylamine (1 mL) under nitrogen, bistributyltin (6 mg; 10 μmol) and Tetrakis(triphenylphosphine)palladium(0) (1 mg; 1 μmol) were added. This reaction mixture was refluxed overnight at 90 °C. The dark yellow crude reaction mixture was purified over a prep silica gel TLC plate using dichloromethane:methanol 9:1 as a solvent. The product was isolated as an oil (Figure 7, **6**) in 25% yield. Mass spectra (ESI) for **6**: 652 (70%), 650 (46%), 648 (44%); [M + Et3N]^+^. NMR (^1^H, 500 MHz) δ ppm in deuterated DMSO: 11.7 (s, 1H), 8.3 (d, 1H, *J* = 7.8 Hz), 8.2 (d, 1H, *J* = 7.6 Hz), 7.90 (d, 1H, *J* = 8.0 Hz), 7.80 (dd, 2H, *J* = 7.6, 1 Hz), 7.52 (d, 1H, *J* = 7.1 Hz), 7.30 (dd, 1H, *J* = 7.8, 1Hz), 1.63–1.56 (m,12H), 1.33 (m, 6H), 0.90 (m, 9H).

A radioiodination hood (CBS Scientific, Inc., San Diego, CA, USA) placed inside a fume hood designated to handle radioactive materials was used to carry out iodine-125 radiolabeling of **6** using our previously reported methods [25,26,27,29]. Iodine-125 sodium iodide was purchased from American Radiolabeled Chemicals, Inc., St. Louis, MO, USA (iodine-125 sodium iodide, carrier-free (specific activity = 643 MBq/μg) in 0.01 N NaOH). The radioiodination of tributyltin derivative **6** was carried out using iodine-125 sodium iodide, similar to the previously described procedure. To tributyltin derivative **6** (0.1 mg in 0.1 mL ethanol), 3.7 MBq [^125^I]NaI in 0.1 mL 0.01 N NaOH was added, followed by the addition of 0.1 mL of 1N HCl. After 2 min of mixing, 0.1 mL of 3% hydrogen peroxide was added, and the mixture was placed on a shaker at room temperature for 60 min. The reaction was terminated by adding 0.1 mL of 0.01N sodium bisulfite solution. After the reaction, the product was extracted with ethyl acetate (3 × 2 mL). The combined ethyl acetate extracts were dried using nitrogen gas, and the residue was purified. The purification and isolation of [^125^I]KuFal184 was carried out on preparative TLC using 9:1 dicholromethane-methanol. RadioTLC (Rf = 0.6) confirmed, with an authentic sample of KuFal184, a radiochemical purity of >95% [^125^I]KuFal184 (Figure 7). Using the molar activity of the commercial no-carrier-added [^125^I]sodium iodide, the molar activity of [^125^I]KuFal184 was estimated to be approximately 90 TBq/mmol. The radiochemical yield of [^125^I]KuFal184 was approximately 25%.

### 4.4. Human Tissue

All postmortem human brain studies were approved by the Institutional Biosafety Committee of the University of California, Irvine. Human postmortem brain tissue samples were obtained from the brain tissue repositories of Banner Sun Health Research Institute, Sun City, AZ, USA, and University of California-Irvine Memory Impairments and Neurological Disorders (UCI MIND) Institute.

### 4.5. In Vitro [^125^I]IPPI Postmortem Human Brain Autoradiography

Human anterior cingulate sections containing corpus callosum were sectioned from the AD subjects. [^125^I]IPPI was used for the binding studies using our previously reported procedures [25]. KuFal184 (1 μM) and MK-6240 (1 μM) were used for competition experiments. Brain sections were air dried, exposed overnight on a phosphor film, and then placed on the Phosphor Autoradiographic Imaging System/Cyclone Storage Phosphor System (Packard Instruments Co., Boonton, NJ, USA). Regions of interest (ROIs) were drawn on the slices, and the extent of binding of [^125^I]IPPI was measured in DLU/mm^2^ using the OptiQuant acquisition and analysis program (Version 4.00.01).

### 4.6. In Vitro [^125^I]KuFal184 Postmortem Human Brain Autoradiography

Human anterior cingulate sections containing corpus callosum were sectioned from the AD subjects. [^125^I]KuFal184 was used for the binding studies for DYRK1A using modifications of [^125^I]IPPI procedures [25]. Brain slices from AD subjects were preincubated in phosphate-buffered saline (PBS) buffer, pH 7.4, for 15 min. Subsequently, this buffer was replaced with fresh PBS buffer containing [^125^I]KuFal184 (2 kBq/mL). KuFal184 (1 μM) was used for competition experiments. The slices were incubated for 60 min at 22 °C. The incubation buffer was removed, and the slices were washed twice (5 min each) with cold PBS buffer, followed by a 2 min rinse with cold water. Brain sections were air dried, exposed overnight on a phosphor film, and then placed on the Phosphor Autoradiographic Imaging System/Cyclone Storage Phosphor System (Packard Instruments Co., Boonton, NJ, USA). Regions of interest (ROIs) were drawn on the slices, and the extent of binding of [^125^I]KuFal184 was measured in DLU/mm^2^ using the OptiQuant acquisition and analysis program (Packard Instruments Co.). For binding studies using urea such as BCU, the incubation buffer contained 10 μM BCU along with [^125^I]KuFal184 (2 kBq/mL). Incubation times and washing was similar to the protocol described above.

### 4.7. Immunohistochemistry

University of California–Irvine, Pathology Services, used Ventana BenchMark Ultra protocols for immunostaining of brain sections using procedures described previously [25].

## 5. Conclusions

In summary, Chimera–AutoDock provided insights into the DYRK1A binding site profile of molecules known to bind to DYRK1A, DYRK1A + MAO-A, and Tau. Binding energies of Tau imaging agents to DYRK1A was found to be moderate. Autoradiographic studies with [^125^I]IPPI confirmed the inability of KuFal184 to affect binding of [^125^I]IPPI. This suggested the lack of DYRK1A binding of [^125^I]IPPI. The selective nature of KuFal184 to DYRK1A is indicative of the usefulness of [^125^I]Kufal184 as an imaging agent. [^125^I]Kufal184 was successfully synthesized and was found to be stable in vitro. Preliminary evaluation of [^125^I]Kufal184 in AD brain slices suggested a need to address membrane permeability issues of [^125^I]Kufal184 because of its anionic nature. The use of urea derivatives appears to assist in increasing the selectivity of [^125^I]Kufal184 binding in AD brain slices. However more studies are planned to establish the binding of [^125^I]Kufal184 to the DA brain slices.

There are several ongoing efforts to develop DYRK1A inhibitors as treatment strategies for AD and DS [42,43]. This effort will be greatly enhanced by the development of selective imaging agents for DYRK1A in order to understand its distribution and activity in neurodegeneration.

## Figures and Tables

**Table 1:** Phosphokinases and Tau phosphosites.

S.No	KINASE	Phosphosites	Plasma and CSF Measured
1	GSK3	Ser46, 184, 198, 199, 202, 210, 214, 235, 237, 258, 262, 285, 289, 305, 324, 352, 356, 396, 400, 404, 409, 413. Thr69, 149, 153, 175, 181, 205, 212, 217, 220, 231, 373	p-Tau181 and 217 (PL) p-Tau181 and 231 (PL) p-Tau181, 217, 231, and 235^3^ (CSF)
2	Cdk5	Ser195, 199, 202, 214, 235, 396, 404. Thr153, 181, 205, 212, 217, 231	p-Tau181 and 217 (PL) p-Tau181 and 231(PL) p-Tau181, 217, 231 and 235 (CSF)
3	P38 MAPK	Ser46, 185, 202, 235, 305, 320, 356, 396, 404, 409, 422. Thr69, 175, 181, 205, 212, 217, 231, 245	p-Tau181 and 217 (PL) p-Tau181 and 231 (PL) p-Tau181, 217, 231, and 235 (CSF)
4	DYRK1A	Ser202, 404; Thr212	none
5	MARK4	Ser262, 356	none
6	Src	Tyr18	none
7	Fyn	Tyr18	none
8	c-Abl	Tyr394	none
9	ROCK	Thr245, 377, Ser409	none

## Data Availability

The data that support the findings of this study are available from the corresponding author upon reasonable request.

## References

[1] Basheer N., Smolek T., Hassan I., Liu F., Iqbal K., Zilka N., Novak P. (2023). Does modulation of tau hyperphosphorylation represent a reasonable therapeutic strategy for Alzheimer’s disease? From clinical studies to the clinical trials. Mol. Psychiatry.

[2] Simrén J. (2021). The diagnostic and prognostic capabilities of plasma biomarkers in Alzheimer’s disease. Alzheimer’s Dement..

[3] Therriault J., Pascoal T.A., Lussier F.Z., Tissot C., Chamoun M., Bezgin G., Servaes S., Benedet A.L., Ashton N.J., Karikari T.K. (2002). Biomarker modeling of Alzheimer’s disease using PET-based Braak staging. Nat. Aging.

[4] Karikari T.K., Pascoal T.A., Ashton N.J., Janelidze S., Benedet A.L., Rodriguez J.L., Chamoun M., Savard M., Kang M.S., Therriault J. (2020). Blood phosphorylated tau 181 as a biomarker for Alzheimer’s disease: A diagnostic performance and prediction modelling study using data from four prospective cohorts. Lancet Neurol..

[5] Thijssen E.H., La Joie R., Wolf A., Strom A., Wang P., Iaccarino L., Bourakova V., Cobigo Y., Heuer H., Spina S. (2020). Diagnostic value of plasma phosphorylated tau181 in Alzheimer’s disease and frontotemporal lobar degeneration. Nat. Med..

[6] Palmqvist S., Janelidze S., Quiroz Y.T., Zetterberg H., Lopera F., Stomrud E., Su Y., Chen Y., Serrano G.E., Leuzy A. (2020). Discriminative Accuracy of Plasma Phospho-tau217 for Alzheimer Disease vs. Other Neurodegenerative Disorders. JAMA.

[7] Janelidze S., Mattsson N., Palmqvist S., Smith R., Beach T.G., Serrano G.E., Chai X., Proctor N.K., Eichenlaub U., Zetterberg H. (2020). Plasma P-tau181 in Alzheimer’s disease: Relationship to other biomarkers, differential diagnosis, neuropathology and longitudinal progression to Alzheimer’s dementia. Nat. Med..

[8] Rodriguez J.L., Karikari T.K., Suárez-Calvet M., Troakes C., King A., Emersic A., Aarsland D., Hye A., Zetterberg H., Blennow K. (2020). Plasma p-tau181 accurately predicts Alzheimer’s disease pathology at least 8 years prior to post-mortem and improves the clinical characterisation of cognitive decline. Acta Neuropathol..

[9] Petersen M.E., Flores-Aguilar L., Head E., Montoliu-Gaya L., Strydom A., Pape S.E., Fortea J., Ashton N.J., Udeh-Momoh C., O’Bryant S.E. (2024). Blood biomarkers in Down syndrome: Facilitating Alzheimer’s disease detection and monitoring. Alzheimer’s Dement..

[10] Deboever E., Fistrovich A., Hulme C., Dunckley T. (2022). The omnipresence of DYRK1A in human diseases. Int. J. Mol. Sci..

[11] Atas-Ozcan H., Brault V., Duchon A., Herault A. (2021). DYRK1A from gene function in development and physiology to dosage correction across life span in Down syndrome. Gene.

[12] Liu T., Wang Y., Wang J., Ren C., Chen H., Zhang J. (2022). DYRK1A inhibitors for disease and therapy: Current status and perspectives. Eur. J. Med. Chem..

[13] Laham A.J., Saber-Ayad M., El-Awady R. (2021). DYRK1A: A down syndrome-related dual protein kinase with a versatile role in tumorigenesis. Cell. Mol. Life Sci..

[14] Liu F., Liang Z., Wegiel J., Hwang Y.-W., Iqbal K., Gurndke-Iqbal I., Ramakrishna N., Gong C.X. (2008). Overexpression of DYRK1A contributes to neurofibrillary degeneration in Down syndrome. FASEB J..

[15] Rammohan M., Harris E., Bhansali R.S., Zhao E., Li L.S., Crispino J.D. (2022). The chromosome 21 kinase DYRK1A: Emerging roles in cancer biology and potential as a therapeutic target. Oncogene.

[16] Ryoo S.-R., Jeong H.K., Radnaabazar C., Yoo J.-J., Cho H.-J., Lee H.-W., Kim I.-S., Cheon Y.-H., Ahn Y.S., Chung S.-H. (2007). DYRK1A-mediated hyperphosphorylation of Tau. J. Biol. Chem..

[17] Kaczmarski W., Barua M., Mazur-Kolecka B., Frackowiak J., Dowjat W., Mehta P., Bolton D., Hwang Y.-W., Rabe A., Albertini G. (2014). Intracellular distribution of differentially phosphorylated dual-specificity tyrosine phosphorylation-regulated kinase 1A (DYRK1A). J. Neurosci Res..

[18] Chaikaud A., Falke H., Krojer T., von Delft F., Arrowsmith C.H., Edwards A.M., Bountra C., Kunick C., Knapp S. (2015). Crystal structure of DYRK1AA in complex with 10-bromo-substituted 11H-indolo [3,2-c]quinolone-6-carboxylic acid inhibitor 5t. Protein Data Bank.

[19] Zheng L., Li Y., Wu D., Xiao H., Zheng S., Wang G., Sun Q. (2023). Development of covalent inhibitors: Principle, design, and application in cancer. Med. Comm. Oncol..

[20] Tarpley M., Oladago H.O., Strepay D., Caligan T.B., Chdid L., Shehata H., Rogues J.R., Thomas R., Laudeman C.P., Onyenwoke R.U. (2021). Identification of harmine and b-carboline analogs from a high throughput screen of an approved drug collection; profiling as differential inhibitors of DYRK1A and monoamine oxidase A and for in vitro and in vivo anti-cancer studies. Eur. J. Pharm. Sci..

[21] Falke H., Chaikuad A., Becker A., Loaec N., Lozach O., Jhaisha S.A., Becker W., Jones P.G., Preu L., Bauman K. (2015). 10-Iodo-11H-indolo[3,2-c]quinoline-6-carboxylic acids are selective inhibitors of DYRK1A. J. Med. Chem..

[22] Shukla R., Kumar A., Kelvin D.J., Singh T.R. (2022). Disruption of DYRK1A-induced hyperphosphorylation of amyloid beta and tau protein in Alzheimer’s disease: An integrative molecular modeling approach. Front. Mol. Biosci..

[23] Meine R., Becker W., Falke H., Preu L., Loaëc N., Meijer L., Kunick C. (2018). Indole-3-Carbonitriles as DYRK1A Inhibitors by Fragment-Based Drug Design. Molecules.

[24] Mazanetz M.P., Fischer P.M. (2007). Untangling tau hyperphosphorylation in drug design for neurodegenerative diseases. Nat. Rev. Drug Discov..

[25] Mukherjee J., Liang C., Patel K.K., Lam P.Q., Mondal R. (2021). Development and evaluation [^125^I]IPPI for tau imaging in post-mortem human Alzheimer’s disease brain. Synapse.

[26] Mondal R., Sandhu Y.K., Kamalia V.M., Delaney B.A., Syed A.U., Nguyen G.A.H., Moran T.R., Limpengco R.R., Liang C., Mukherjee J. (2023). Measurement of Ab amyloid and Tau protein in postmortem human Alzheimer’s disease brain by autoradiography using [^18^F]flotaza, [^125^I]IBETA and [^124/125^I]IPPI and immunohistochemistry analysis using QuPath. Biomedicines.

[27] Balint B., Weber C., Cruzalegui F., Burbridge M., Kotschy A. (2017). Structure-based design and synthesis of harmine derivatives with different selectivity profiles in kinase versus monoamine oxidase inhibition. ChemMedChem.

[28] Liang C., Paclibar C.G., Gonzaga N.L., Sison S.A., Bath H.S., Biju A.P., Mukherjee J. (2024). [^125^I]IPC-Lecanemab: Synthesis and evaluation of Ab-plaque-binding antibody and comparison with small-molecule [^18^F]Flotaza and [^125^I]IBETA in postmortem human Alzheimer’s disease. Neurol. Int..

[29] Hostetler E.D., Walji A.M., Zeng Z., Miller P., Bennacef I., Salinas C., Connolly B., Gantert L., Haley H., Holahan M. (2016). Preclinical characterization of 18F-MK-6240, a promising PET tracer for in vivo quantification of human neurofibrillary tangles. J. Nucl. Med..

[30] Limpengco R.R., Liang C., Sandhu Y.K., Mukherjee J. (2023). [^125I^]INFT: Development and Evaluation of a new Tau Imaging agent in Post-Mortem Human Alzheimer’s disease brain. Molecules.

[31] Nguyen G.A.H., Liang C., Mukherjee J. (2022). [^124^I]IBETA, a new Ab amyloid plaque PET imaging agent for Alzheimer’s disease. Molecules.

[32] Sison S.A., Paclibar C.G., Liang C., Mukherjee J. (2024). Radioiodinated tau imaging agent III molecular modeling, synthesis, and evaluation of a new tau imaging agent, [^125^I]ISAS in post-mortem human Alzheimer’s disease brain. Molecules.

[33] Salam R., Chowdhury S.M., Marshall S.R., Gneid H., Busschaert N. (2021). Increasing membrane permeability of carboxylic acid-containing drugs using synthetic transmembrane anion transporters. ChemComm.

[34] Braak H., Thal D.R., Ghebremedhin E., Tredici K.D. (2011). Stages of the pathologic process in Alzheimer’s disease age categories from 1 to 100 years. J. Neuropathol. Exp. Neurol..

[35] Cools R., Kerkhofs K., Leitao R.C.F., Bormans G. (2023). Preclinical evaluation of novel PET probes for dementia. Sem. Nucl. Med..

[36] Cao L., Kong Y., Ji B., Ren Y., Guan Y., Ni R. (2021). Positron emission tomography in animal models of taupathies. Front. Aging Neurosci..

[37] Groot C., Villeneuve S., Smith R., Hansson O., Ossenkoppele R. (2022). Tau PET imaging in neurodegenerative disorders. J. Nucl. Med..

[38] Shah N., Ghazaryan N., Gonzaga N.L., Paclibar C.G., Biju A.P., Liang C., Mukherjee J. (2024). Glutamate’s effects on the N-methyl-D-aspartate (NMDA) receptor ion channel in Alzheimer’s disease brain: Challenges for PET radiotracer development for imaging the NMDA ion channel. Molecules.

[39] Syed A.U., Liang C., Patel K.K., Mondal R., Kamalia V.M., Moran T.R., Ahmed S.T., Mukherjee J. (2023). Comparison of Monoamine oxidase-A, Ab plaques, Tau and Translocator protein in postmortem human Alzheimer’s disease brain. Int. J. Mol. Sci..

[40] Sandhu Y.K., Bath H.S., Shergill J., Liang C., Syed A.U., Ngo A., Karim F., Serrano G.E., Beach T.G., Mukherjee J. (2024). [^18^F]Flotaza for Aβ plaque diagnostic imaging: Evaluation in postmortem human Alzheimer’s disease brain hippocampus and PET/CT imaging in 5xFAD transgenic mice. Int. J. Mol. Sci..

[41] Zanderigo F., D’Agostino A.E., Josh N., Schain M., Kumar D., Parsey R.V., DeLorenzo C., Mann J.J. (2018). [^11^C]Harmine binding to brain monoamine oxidase A: Test-retest properties and noninvasive quantification. Mol. Imag. Biol..

[42] Meijer L., Chretien E., Ravel D. (2024). Leucettinib-21, a DYRK1A kinase inhibitor as clinical drug candidate for Alzheimers disease and Down syndrome. J. Alzheimer’s Dis..

[43] Stringer M., Goodlett C.R., Roper R.J. (2017). Targeting trisomic treatments: Optimizing Dyrk1a inhibition to improve Down syndrome deficits. Mol. Genet. Genom. Med..

